# Tsunami disaster resilience through dramatisation of local wisdom: The performance of Nandong Smong by Acehnese high school students in Simeulue regency, Indonesia

**DOI:** 10.4102/jamba.v18i1.1978

**Published:** 2026-03-23

**Authors:** Daska Aziz, Suhendro Suhendro, Novia Zalmita, Cut Vita R. Jummi, M. Hafizul Furqan

**Affiliations:** 1 Department of Geography Education, Faculty of Teacher Training and Education, Universitas Syiah Kuala, Banda Aceh, Indonesia; 2 Department of Geography Education, Faculty of Social Sciences Education, Universitas Pendidikan Indonesia, Bandung, Indonesia

**Keywords:** drama, resilience, Nandong Smong, tsunami, local wisdom

## Abstract

Nandong Smong represents a vital element of Aceh’s local cultural wisdom that has long contributed to community-based tsunami risk reduction. Following the devastating 2004 Indian Ocean tsunami, disaster mitigation initiatives have been integrated into school curricula, with innovative pedagogical approaches gaining prominence. This study explores the use of drama, specifically role-playing, to express and revitalise Nandong Smong as a medium for strengthening tsunami disaster resilience among high school students. An experimental design employing a pre-test–post-test group and a quantitative analytical approach was used to assess the effectiveness of the intervention. The results demonstrate that the role-playing model significantly improved students’ tsunami disaster resilience, with a contribution rate of 57.54%. The results highlight the potential for integrating local wisdom into creative learning strategies and suggest that similar approaches can be adopted in other disaster-prone coastal schools to enhance preparedness and resilience.

**Contribution:**

The contribution of this research lies in its innovative integration of Nandong Smong local wisdom with drama-based role-playing learning as a practical non-structural mitigation strategy for high school students. This study demonstrates how culturally rooted narratives can be transformed into interactive learning experiences that enhance disaster awareness, preparedness and adaptive capacity among vulnerable groups in schools. Bridging traditional knowledge with contemporary pedagogical methods, this study provides a transferable framework that can be adapted to other tsunami-prone coastal communities. The findings highlight the potential of transformative, culturally responsive educational approaches to strengthen community resilience and sustain intergenerational disaster knowledge.

## Introduction

Global fluctuations in disaster frequency and intensity have promoted the international community to adopt proactive measures to strengthen disaster resilience (Fazeli et al. 2024). Conceptually known as disaster risk reduction (DRR), this effort, within the Sendai Framework, reflects a global policy shift from reactive disaster response to a more holistic approach that emphasises the identification, assessment and reduction of risk through an understanding of vulnerability, exposure, capacity and efforts to strengthen resilience (Aitsi-Selmi et al. 2015; UNDRR 2015). Within this effort, the education plays a pivotal role in cultivating risk awareness and adaptive capacities from an early age (Lin & Lee 2025; Rahma, Mardiatno & Hizbaron 2024).

Effective disaster education extends beyond theoretical knowledge to encompass experiential and participatory learning, such as simulations, evacuation drills and the transmission of cross-generational experiences, which collectively enhance preparedness and responsiveness (Grau Vila 2024). Schools, therefore, function not only as centres of learning but also as vital hubs for disseminating disaster-related knowledge and resilience practices to families and the broader community throughout the mitigation, response and recovery phases (Cels et al. 2023; Kawasaki et al. 2022; Pan 2023).

A key strength of DRR education lies in its potential to foster resilience among children and adolescents, who are often among the most vulnerable in hazard-prone environments (Kyambade, Sewante & Namatovu 2025; McPherson & Casanueva Baptista 2024). Empirical studies show that young people can acquire survival skills through contextual, engaging and participatory approaches – skills that not only support their own safety but also contribute positively to their households’ disaster preparedness (Harada, Shoji & Takafuji 2023; Hoque et al. 2021). Approaches incorporating drama, role-play and real-life simulations have been demonstrated to increase disaster literacy, enhance adaptive capacity and strengthen students’ emotional and social resilience (Bubeck et al. 2024; Di Bucci, Dolce & Santini 2025). In this process, teachers serve as both facilitators of participatory learning and influential resilience leaders within their communities (Fu & Zhang 2024).

In Indonesia, particularly within local and indigenous communities, traditional knowledge has long served as an effective foundation for disaster resilience. A well-known example is ‘Smong’, an oral tradition from the Simeulue Island community that enabled residents to recognise early tsunami signs and evacuate effectively during the 2004 Indian Ocean tsunami, resulting in remarkably low casualty rates (Rahman et al. 2024). The term *Smong*, derived from the Devayan language, describes the sequence of shaking ground, receding seawater and the arrival of giant waves and urges immediate evacuation to higher ground (Rahman et al. 2022). As a form of indigenous knowledge, Smong represents a critical asset for DRR, bridging historical experiences and future preparedness within disaster education (Pisa 2024; Suarmika et al. 2022; Tavares, Alves & Vásquez 2021; Trogrlić et al. 2021; Widuri et al. 2023). Consistent with Kinol et al. (2023), localised and culturally embedded communication tends to resonate more deeply than generic global messages, as it connects with people’s lived experiences and local realities.

**FIGURE 1:**
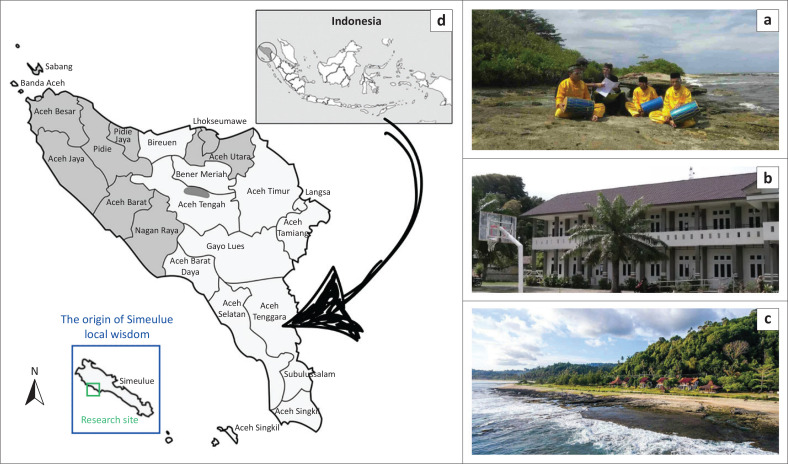
(a) Local wisdom Nandong Smong, (b) Research site, (c) Simeulue Island and (d) Location of SHS 1 Salang on Simeulue Island.

Despite these insights, existing studies often treat indigenous knowledge and participatory pedagogical approaches as separate domains or view students primarily as passive recipients rather than active contributors to resilience-building processes. Few studies have explicitly integrated local cultural narratives, performing arts and student participation within a comprehensive disaster learning model. This gap highlights the need to explore how local wisdom-based participatory theatre, such as Smong Nandong (Figure 1), can serve as an educational medium that engages students cognitively, emotionally, socially and behaviourally in strengthening disaster resilience.

Integrating Smong into learning activities through performing arts, particularly participatory drama, offers a meaningful way to deepen student engagement and understanding. Drama does more than transmit information; it fosters empathy, encourages behavioural transformation and supports the development of shared awareness and collective responsibility towards disaster risks (Brown et al. 2017; Busby et al. 2024; Khusna et al. 2023). Against this backdrop, the present study aims to: (1) develop Nandong Smong drama guidelines and (2) evaluate the effectiveness of their implementation in enhancing tsunami disaster resilience among high school geography students in Aceh, Indonesia.

## Research design and methods

This study aims to examine the level of disaster resilience among high school students in Simeulue regency, Aceh Province, Indonesia and to evaluate the effectiveness of integrating *Nandong Smong*, a well-known form of local wisdom used to recognise and respond to tsunamis, into classroom learning through drama-based role-play.

**FIGURE 2:**
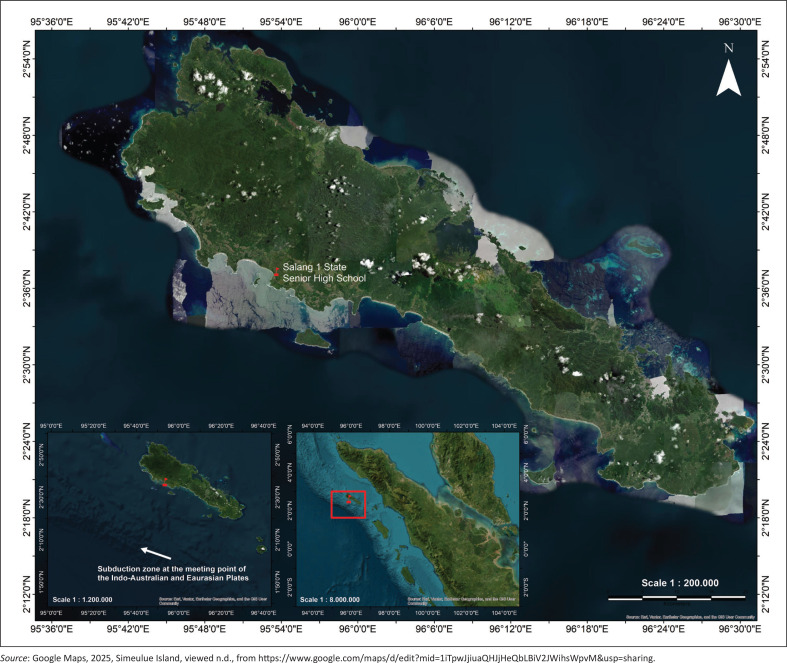
Senior High School 1 Salang, Simeulue regency, Aceh.

This research was conducted at a secondary school on the coast of Simeulue Island (SHS 1 Salang, Simeulue regency, Aceh) (Figure 2). In terms of geotectonics, this region is located in an earthquake and tsunami-prone zone because of tectonic activity between the Indo-Australian and Eurasian plates. Historically, the threat of tsunamis has not only caused safety concerns but has also significantly impacted local livelihoods. Some communities rely on fishing, coastal agriculture and trading activities, which are highly vulnerable to tsunamis. This situation places vulnerable groups, such as children and their families, in a layered risk environment, emphasising the need for contextual interventions through education on sustainable DRR, including the preservation of local wisdom such as role-playing games at school.

A quantitative experimental research design was adopted, employing a one-group pre-test–post-test design to measure changes in students’ disaster resilience before and after the intervention. This design was chosen to determine the effectiveness of the pedagogical strategy while allowing for controlled assessment of learning outcomes.

The study involved 20 high school students who were selected using purposive sampling. The sampling decision was based on the relevance of the geography curriculum content – particularly topics related to disaster mitigation – to ensure that participants had appropriate baseline exposure to the subject matter. The selected school is situated within Simeulue regency, the cultural origin of *Nandong Smong*, making it a suitable context for examining the integration of local wisdom into disaster education.

Inclusion criteria were applied in determining the sample and controlling participant variation. These criteria included students at SHS 1 Salang at the same grade level and of relatively homogeneous ages. Meanwhile, the sample size was determined using a purposive sampling approach, taking into consideration contextual appropriateness and subject accessibility, such as geography subjects. To ensure that all participants were of comparable ability, a pre-test was conducted before the intervention. Although the participants came from a relatively homogeneous environment, individual variations in experience of the tsunami were recognised, representing the real conditions of coastal communities living with the risk of tsunamis.

The intervention consisted of a structured learning activity in which students learned about *Nandong Smong* through role-playing and drama performance techniques. The educational drama was developed based on a literature review of local cultural narratives, historical accounts of past tsunami events and existing studies on the role of *Nandong Smong* in community disaster preparedness. The role-play activity allowed students to enact scenarios related to tsunami recognition, early warning and response behaviours embedded within the tradition.

Data on students’ disaster resilience were collected using a 25-item validated test instrument, designed to assess key dimensions such as hazard knowledge, preparedness behaviour, risk perception and decision-making during disaster events. The instrument underwent content validation by experts in disaster education and was piloted to ensure clarity and reliability prior to administration. To test the validity of questions, a formula is needed, specifically the product-moment correlation formula. The validity of the instrument questions was tested on 20 respondents outside the research sample (Table 1).

**TABLE 1:** Instrument measurement validation results.

Item	*r*-table	*r*-value	Describe
1	0.44	0.4860	Item Valid
2	0.44	0.5330	Item Valid
3	0.44	0.5400	Item Valid
4	0.44	0.4590	Item Valid
5	0.44	−0.1200	Item No Valid
6	0.44	0.5130	Item Valid
7	0.44	−0.4500	Item No Valid
8	0.44	0.5330	Item Valid
9	0.44	0.6920	Item Valid
10	0.44	0.6935	Item Valid
11	0.44	0.6720	Item Valid
12	0.44	0.3740	Item No Valid
13	0.44	0.8100	Item Valid
14	0.44	0.6940	Item Valid
15	0.44	0.7311	Item Valid
16	0.44	0.4940	Item Valid
17	0.44	0.6940	Item Valid
18	0.44	0.3130	Item No Valid
19	0.44	0.6820	Item Valid
20	0.44	0.4150	Item No Valid
21	0.44	0.6920	Item Valid
22	0.44	0.4940	Item Valid
23	0.44	0.6940	Item Valid
24	0.44	0.6060	Item No Valid
25	0.44	0.6060	Item Valid

Refer to the map of the Simeulue coastal area presented earlier. According to the local wisdom of Smong Nandong, the community is taught to quickly move away from the coast to higher ground after a strong earthquake and when seawater recedes. If a village on the map is about 300 m from the shoreline and has two evacuation hills, the most effective mitigation strategy in line with Smong Nandong is:

- Await official instructions from the government before starting the evacuation.
- Guide the evacuation towards the nearby hill, even though the route is narrower.
- Gather villagers for data collection prior to the evacuation.
- Conduct a horizontal evacuation along the coast to prevent congestion.
- Use motor vehicles for mass evacuation to the city.

Next, we analysed Table 1, which shows an r-hitung value of 0.458. This value was then compared with the *r*-table value, calculated based on the number of respondents at a 5% significance level, which is 0.444.

The pre-test was administered before the intervention, and the post-test was conducted immediately after the role-play activity to measure learning gains attributable to the drama-based integration of *Nandong Smong*. In this study, we carefully examined the Hawthorne effect, which is the tendency of participants to modify their behaviour and enhance their performance because they know they are being observed in a research setting. To reduce the impact of this effect, we conducted the activity in a regular learning environment without informing participants of the intervention, allowing their natural behaviour to be observed. Additionally, the teacher’s instructions, such as that student participation would not influence academic assessment, helped to lessen psychological pressure and behavioural bias. Through this approach, we believe we successfully minimised the Hawthorne effect.

Both descriptive and inferential quantitative analyses were employed. Descriptive statistics (means, standard deviations and percentage scores) were used to present the distribution and general trend of students’ disaster resilience levels. Inferential analysis was conducted to test the effectiveness of the intervention. A paired comparison of pre-test and post-test scores was used to determine whether the role-play learning strategy significantly improved students’ disaster resilience. All analyses were conducted using appropriate statistical software.

Additionally, qualitative insights from literature studies informed the contextualisation of *Nandong Smong* within the educational drama but were not used as a primary data source.

### Ethical considerations

Ethical clearance to conduct this study was obtained from the Research Ethics Committee of Syiah Kuala University Banda Aceh (No. 156/UN11.F13/KM/DPIPS/2025).

## Results

### Designing Nandong Smong through drama for tsunami disaster resilience

The integration of *Nandong Smong* into a drama-based learning model positions children not merely as recipients of disaster knowledge but as active creators and interpreters of local wisdom. The participatory design blends cultural narratives, intergenerational and experiential learning, allowing the *Smong* myth, long used by coastal Acehnese communities as a marker of tsunami warning signs, to become a dynamic pedagogical tool. Narrative-based learning of this kind offers a simple but powerful strategy for addressing complex societal challenges, as stories can shape understanding, memory and collective identity (Canlas & Kazakbaeva 2023; Saari & Mullen 2022). Consistent with Busby et al. (2024), the findings show that applied theatre can motivate shifts in knowledge, attitudes and behaviour relevant to disaster preparedness.

The drama structure deliberately stimulates ‘Episodes of Situated Learning’ (Piangiamore & Maramai 2024), where children learn in contextually meaningful situations. By assuming roles such as survivors, first responders or traditional community leaders, participants actively reorganise information about tsunamis into stronger and more accessible memory structures (De Guttry & Ratter 2022). This process encourages not only cognitive understanding of tsunami hazards but also emotional engagement and empathetic appreciation of community experience. Indeed, the *Nandong Smong* tradition itself – described by Sobary in Wani (2020) –functions as a cultural buffer, offering psychological strength, confidence and a sense of continuity as communities face recurring earthquake and tsunami risks.

**FIGURE 3:**
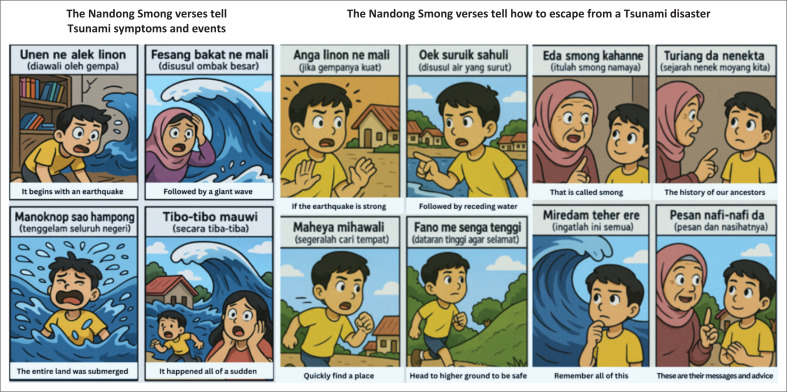
The Nandong Smong Verses.

To illustrate these functions, Figure 3 visualises key verses documented by Desfandi (2019), which convey both the early signs of a tsunami and the steps required for survival. These verses reinforce local knowledge systems, and the original *Nandong Smong* performance can be accessed for reference (https://www.youtube.com/watch?v=OpF-G078LZ4).

### Revitalising local narratives through children’s drama

The findings also indicate that embedding *Nandong Smong* into drama serves as a counterbalance to externally driven disaster education interventions, which frequently marginalise or overwrite local perspectives (Azad, Haque & Choudhury 2022; Choudhury et al. 2021). Through dramatisation, children reconstruct stories rooted in their own communities, curated with the support of teachers and facilitators. This positions the drama as both a reflective and generative practice – one that preserves local wisdom while enabling children to reinterpret it in contemporary contexts. Such processes resonate with Koopman’s (2023) argument that reviving localised knowledge can strengthen community resilience.

Moreover, the drama-based approach fosters social learning and strengthens community-based risk communication (Bubeck et al. 2024). As students work collaboratively, they build interpersonal relationships, enhance mutual support and practice coordinated responses – elements shown to be beneficial for children’s disaster awareness and readiness (Amri et al. 2022; Suarmika et al. 2022; Wang et al. 2022). Within the school environment, drama provides a safe, structured and familiar space for practicing response behaviours, ultimately reinforcing preparedness capacities (Harada et al. 2023; Takefuji 2023).

Overall, the results demonstrate that drama is not only a tool for cultural expression but also a transformative strategy for disaster education. By integrating local wisdom, emotional engagement and embodied learning, the *Nandong Smong* drama model strengthens both knowledge retention and community-rooted resilience among Acehnese high school students.

### Successful interventions in improving tsunami disaster resilience

Schools play a critical role in shaping disaster literacy from an early age, particularly because children constitute one of the most vulnerable groups during disaster events (Hoque et al. 2021; Sarafova 2023).

However, strengthening children’s readiness requires adaptive and culturally responsive pedagogical approaches. Techniques such as the use of narrative-based learning, educational play and drama have been shown to enhance cognitive engagement and awareness. For instance, drama-based interventions within the Costa Resiliente programme in Southern Chile demonstrated positive gains in students’ hazard comprehension and preparedness behaviours (Villagra et al. 2023).

Similarly, Howard (2023) highlights that the educational systems can nurture social awareness and community resilience when learning materials reflect local knowledge systems and students’ lived realities.

Integrating local narratives such as the *Nandong Smong* tradition in Simeulue into classroom learning provides an immersive experience that communicates both information and emotional urgency (Hanifa & Yusra 2023; Li & Li 2021). When disaster materials are contextualised, students not only acquire factual knowledge but also develop empathy, situational understanding and a sense of personal responsibility towards DRR (Hanifa et al. 2024; Makrooni et al. 2025). Such contextual DRR education helps cultivate deeper, more active learning outcomes grounded in local wisdom.

### Improvement in knowledge scores after the intervention

**FIGURE 4:**
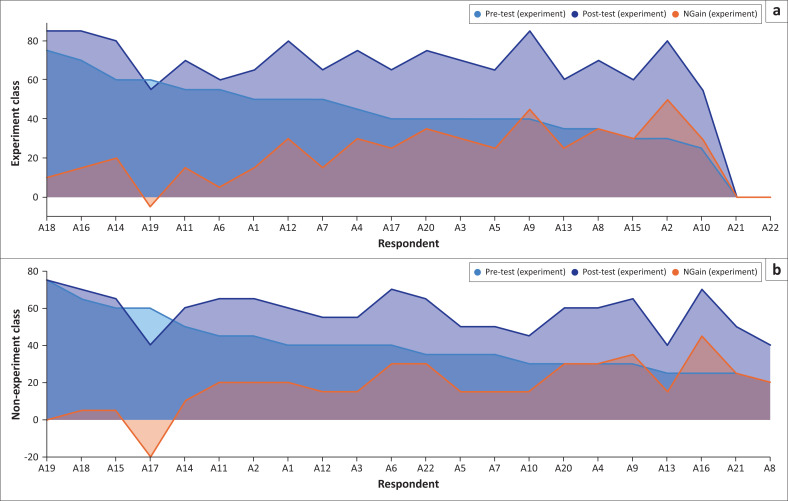
Graph of intervention results.

The intervention produced a clear difference between pre-test and post-test scores, demonstrating significant individual improvement following the *Nandong Smong* drama-based activity. As shown in Figure 4, the majority of participants achieved higher post-test scores, indicating strengthened understanding of tsunami indicators, preparedness procedures and early evacuation cues embedded in the *Smong* narrative (Figure 4).

Although a smaller number of learners recorded stagnant or slightly lower scores, this did not alter the overall positive trend, aligning with similar findings where culturally grounded pedagogies enhanced DRR learning outcomes (Rahman et al. 2024).

A notable shift was observed in the distribution of scores; while pre-test performance clustered around the low-to-moderate range, post-test scores concentrated in the upper-intermediate band. This suggests that the intervention not only enhanced individual comprehension but also reduced disparities in understanding among students. In disaster education, such equalisation is crucial because it reflects more equitable preparedness among vulnerable groups, including school-aged children (Hoque et al. 2021; Pennea et al. 2021).

### Changes in tsunami disaster resilience scores

**FIGURE 5:**
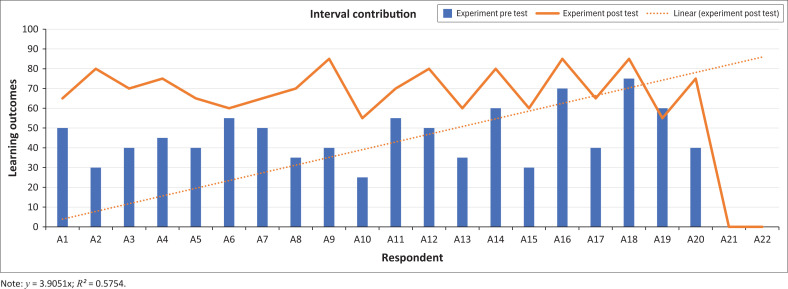
Graph of tsunami disaster resilience intervention results.

Figure 5 illustrates an overall upward trend in tsunami disaster resilience following the intervention. The linear trend (*R*^2^ ≈ 57.54) indicates a moderate but consistent increase in resilience indicators, such as awareness of evacuation routes, confidence in responding to warnings and understanding of safe behaviours.

While the improvement is not drastic, it is steady and meaningful. A few instances of reduced post-intervention scores may reflect temporary psychosocial factors, insufficient time for absorption of new material or the need for more differentiated instructional strategies. Previous research similarly emphasise that experiential and theatre-based learning must be supported by adequate school resources, psychosocial facilitation and structured follow-up sessions (Rezaei & Mohammadinia 2025).

### Sustaining the gain of the intervention

The overall findings underscore that drama-based, culturally embedded DRR education, such as performing *Nandong Smong*, has significant potential to improve both disaster knowledge and resilience among high school students. However, long-term effectiveness depends on the continuity and institutionalisation of such practices. Persistent barriers remain, including limited teacher capacity, time constraints and misalignment between national curricula and local cultural knowledge (Park, Lim & Song 2024). Strengthening these interventions requires comprehensive teacher training, ongoing development of contextual learning materials (Bergström, Norberg & Nordlund 2023; Hanifa et al. 2024) and supportive education policies that formally integrate local wisdom into DRR pedagogy (Cvetković, Nikolić & Lukić 2024; Seddighi et al. 2023; Ismail et al. 2024).

Overall, the results affirm that when local knowledge such as *Nandong Smong* is embedded within creative educational approaches like drama, disaster learning evolves beyond simple information transfer. It nurtures a generation of learners who are not only informed but also resilient, culturally grounded and better prepared to navigate future uncertainties.

## Discussion

The principle that ‘the past teaches the future’ served as the philosophical foundation for designing Nandong Smong as a pedagogical medium for disaster resilience education. This concept resonates with the principle of uniformitarianism, proposed by Hutton and Lyell, which posits that present-day processes can illuminate historical events (Piangiamore & Maramai 2024). Within the disaster context, this perspective is particularly relevant because the historical experience of the Simeulue community in responding to tsunamis has been preserved through the oral tradition of *Smong*. Today, these narratives are not only remembered but are actively transmitted through educational settings, including drama-based learning. Thus, history becomes more than a chronological record; it functions as a contextually effective pedagogical tool that supports the internalisation of disaster knowledge (Koopman 2023).

Schools occupy a central role in building disaster resilience, functioning not only as learning environments but also as key social institutions in post-disaster recovery and transformation (Cels et al. 2023; Johnston, Taylor & Ryan 2022). Yet the contribution of schools to disaster mitigation and capacity building is often undervalued (Pal et al. 2023). Teacher involvement is therefore crucial in cultivating student resilience (Parrott et al. 2025). However, existing studies demonstrate that disaster education in schools often remains predominately cognitive, lacking experiential components such as simulations and practical exercises (Asiah et al. 2023; Maryani 2021). In response, the integration of Nandong Smong into drama represents a strategic educational innovation that engages students cognitively, affectively and psychomotorically.

The findings indicate that the participatory, drama-based intervention contributed meaningfully to enhancing students’ understanding of tsunami hazards and their overall disaster resilience. The increase in post-test scores relative to pre-test scores among most participants demonstrates individual progress and aligns with Bubeck et al. (2024), who argue that participatory theatre is an effective tool for risk communication, especially when embedded within community-based approaches. Although a few students exhibited stagnation or decline, the overall upward trend supports the value of participatory methodologies in disaster education.

**FIGURE 6:**
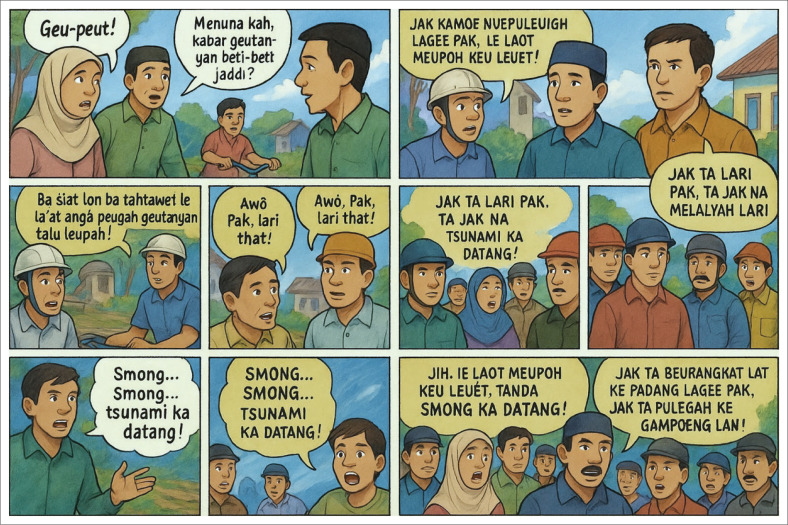
Examples of panels in the Nandong Smong drama script.

The initial pre-test score distribution, which skewed low, further highlights the limited baseline understanding of disaster concepts among students, particularly regarding tsunamis. The rightward shift in the post-test distribution illustrates not only increased understanding but also a more uniform learning outcome across participants. This outcome suggests that the intervention successfully accommodated varying levels of prior knowledge, a finding consistent with Di Bucci et al. (2025), who emphasise the contribution of role-play to strengthening DRR competencies. Figure 6 presents an example of how the Nandong Smong script was visualised and integrated into the drama intervention.

While the increase in scores was steady rather than dramatic, with a slight trendline slope and a low R^2^ value, these variations underscore the diverse ways students respond to the same learning stimulus. Such disparities reflect the influence of contextual factors – including family support, information access and school facilities – on individual learning trajectories, as discussed by Rezaei and Mohammadinia (2025). These findings highlight the necessity of tailoring disaster education to local contexts and student needs, rather adopting uniform instructional models.

More broadly, the results reinforce the importance of participatory and culturally grounded approaches in disaster education. Scholars such as Azad et al. (2022) and Choudhury et al. (2021) emphasise that external interventions frequently overlook local voices, knowledge and heritage. A drama-based approach, anchored in local wisdom such as Nandong Smong, does more than convey cognitive information – it activates emotional engagement, empathy and social cohesion, consistent with Busby et al. (2024) on the transformative potential of applied theatre.

Therefore, effective disaster education must move beyond cognitive delivery to incorporate creative, experiential and culturally rich methods. In doing so, it fosters not only individual competencies but also collective resilience. The study demonstrates that integrating local cultural expressions into school-based programming contributes both to preserving heritage and to building disaster-ready communities. Ultimately, Nandong Smong as a drama affirms the synergistic relationship between cultural continuity and disaster resilience, ensuring that vital local wisdom persists while equipping younger generations with practical skills for future hazards. This study has limitations, such as the number of participants being limited to only one school, even though there are 25 high schools on Simeulue Island. Therefore, we recommend implementing this role model in all schools to achieve more definitive results.

## Conclusion

This study demonstrates that Nandong Smong participatory drama approach can effectively enhance tsunami disaster resilience among Acehnese high school students. By engaging learners in the collective creation and performance of drama grounded in local wisdom, students developed deeper and more meaningful understanding of the cultural concept of the ‘Smong’. They were not only able to explain its significance but also to clearly articulate appropriate actions to take during a tsunami threat. This indicates that drama-based learning serves as a powerful medium for cultivating practical preparedness skills that students can apply in everyday life.

The findings further show that participatory drama fosters more than just cognitive learning. It enables learners to identify early warning signs, build confidence and strengthen their capacity for coordinated response. While the intervention is rooted in culturally familiar narratives and expressions, it promotes learning that is contextually relevant, emotionally resonant and sustainable. Such culturally anchored approaches align closely with the study’s objective of developing disaster education models that effectively strengthen resilience in tsunami-prone settings.

Overall, this research confirms that Nandong Smong is not only a valuable cultural heritage but also a potent educational tool for shaping disaster-resilient youth. Integrating local wisdom with participatory pedagogies has demonstrated significant potential in nurturing resilience that is enduring and embedded in students’ understanding, attitudes and behaviours (Winkens, Lemke & Leicht-Scholten 2024). These results underscore the promise of replicating similar culturally grounded disaster education initiatives in other coastal communities that possess their own local disaster narratives.
